# Colibactin leads to a bacteria-specific mutation pattern and self-inflicted DNA damage

**DOI:** 10.1101/gr.279517.124

**Published:** 2024-08

**Authors:** Emily Lowry, Yiqing Wang, Tal Dagan, Amir Mitchell

**Affiliations:** 1 Department of Systems Biology, University of Massachusetts Chan Medical School, Worcester, Massachusetts 01605, USA;; 2 Institute of General Microbiology, Kiel University, 24118 Kiel, Germany

## Abstract

Colibactin produced primarily by *Escherichia coli* strains of the B2 phylogroup cross-links DNA and can promote colon cancer in human hosts. Here, we investigate the toxin's impact on colibactin producers and on bacteria cocultured with producing cells. Using genome-wide genetic screens and mutation accumulation experiments, we uncover the cellular pathways that mitigate colibactin damage and reveal the specific mutations it induces. We discover that although colibactin targets A/T-rich motifs, as observed in human colon cells, it induces a bacteria-unique mutation pattern. Based on this pattern, we predict that long-term colibactin exposure will culminate in a genomic bias in trinucleotide composition. We test this prediction by analyzing thousands of *E. coli* genomes and find that colibactin-producing strains indeed show the predicted skewness in trinucleotide composition. Our work reveals a bacteria-specific mutation pattern and suggests that the resistance protein encoded on the colibactin pathogenicity island is insufficient in preventing self-inflicted DNA damage.

Competitive interactions are common in microbial communities, including the human gut microbiome (Kern et al. 2021). Secreted toxins that target neighboring microbes are a common mechanism underlying such competitive interactions. Colibactin is a bacteria-secreted genotoxin that can bind DNA in neighboring cells (Nougayrède et al. 2006; Vizcaino and Crawford 2015; Pleguezuelos-Manzano et al. 2020). Colibactin damage is toxic for some bacteria species (Chen et al. 2022; Silpe et al. 2022; Wong et al. 2022) and can also harm the host intestinal cells (Dziubańska-Kusibab et al. 2020; Pleguezuelos-Manzano et al. 2020). Colibactin-producing bacteria have been associated with multiple human diseases ranging from inflammatory bowel disease to colorectal cancers (Buc et al. 2013; Eklöf et al. 2017; Dejea et al. 2018; Iyadorai et al. 2020). Although colibactin-induced damage in host cells is extensively investigated owing to its clinical relevance, its toxicity to bacteria remains underexplored. Addressing this knowledge gap could elucidate how colibactin impacts the host microbiome and whether colibactin expression is associated with any burden on cells producing the toxin. We examined multiple aspects of colibactin toxicity in *Escherichia coli*. We discovered that although colibactin acts as a mutagen and thus depends on a similar mechanism of action in both bacterial and mammalian systems, fundamental differences in bacteria exist.

Colibactin is encoded by a 54 kb genomic region known as the *pks* island. This region comprises 19 genes needed to synthesize and export the toxin, including nonribosomal peptide synthetases and polyketide synthases (Nougayrède et al. 2006). The island also encodes a cyclopropane hydrolase (*clbS*) that protects colibactin-producing cells from the toxin (Nougayrède et al. 2006; Bossuet-Greif et al. 2016; Tripathi et al. 2017). The toxin itself contains two cyclopropane rings that alkylate DNA and cause interstrand cross-links (Vizcaino and Crawford 2015; Tripathi et al. 2017; Bossuet-Greif et al. 2018; Xue et al. 2018, 2019; Wilson et al. 2019). Colibactin is most commonly found in *E. coli* strains of the B2 phylogroup and is expressed by both pathogenic and commensal strains (Nougayrède et al. 2006; Wami et al. 2021). Bacteria harboring the *pks* island are estimated to exist in the gut microbiome of 20%–30% of healthy individuals (Nougayrède et al. 2006; Dubois et al. 2010; Eklöf et al. 2017; Dejea et al. 2018; Dubinsky et al. 2020; Watanabe et al. 2020). The prevalence of *pks*-positive bacteria increases to ∼60% in patients with colorectal cancer and inflammatory bowel disease (Buc et al. 2013; Eklöf et al. 2017; Dejea et al. 2018; Dubinsky et al. 2020).

The clinical relevance of colibactin in various human diseases underlies the widespread efforts to study its toxicity in mammalian models. In vitro experiments showed that colibactin causes DNA damage and leads to cell-cycle arrest in various mammalian cells. This damage requires contact between bacteria and the targeted host cells (Nougayrède et al. 2006; Bossuet-Greif et al. 2018; Reuter et al. 2018; Silpe et al. 2022; Wong et al. 2022). Colibactin interstrand cross-links are resolved through the activation of multiple DNA repair pathways, including the nonhomologous end-joining (Cuevas-Ramos et al. 2010), homologous recombination (Dougherty et al. 2023), and the Fanconi anemia repair pathways (Bossuet-Greif et al. 2018; Dougherty et al. 2023). Because repair of DNA cross-links introduces double-stranded breaks, they are potentially mutagenic, leading to the hypothesis that colibactin may predispose individuals to colon cancer (Cuevas-Ramos et al. 2010). In human cells, colibactin-induced mutations are commonly found in hexameric A/T-rich DNA motifs (Dziubańska-Kusibab et al. 2020; Pleguezuelos-Manzano et al. 2020). This enrichment is attributed to the particularly narrow minor groove of these sequences, which promotes colibactin binding (Dziubańska-Kusibab et al. 2020). Recent work has found that a colibactin-associated mutational signature is detected in 5%–10% of colorectal cancers (Pleguezuelos-Manzano et al. 2020), supporting its involvement in cancer.

Colibactin toxicity has also been observed in bacteria. Auto-toxicity was observed in colibactin-producing bacteria genetically engineered to knockout *clbS* (the protective cyclopropane hydrolase) and became more pronounced upon inactivation of the nucleotide excision repair pathway (Bossuet-Greif et al. 2016; Tripathi et al. 2017). Studies also showed colibactin can target other bacterial species, including several *Staphylococcus* species (Chen et al. 2022; Wong et al. 2022), several *Vibrio* species, *Clostridium difficile*, and *Enterobacter aerogenes* (Chen et al. 2022). A recent study suggested that colibactin damage arises from prophage induction (Silpe et al. 2022). However, prophage-cured *S. aureus* remain susceptible to colibactin, indicating that toxicity mechanisms beyond prophage-induction exist (Wong et al. 2022). Despite the multiple reports of colibactin damage in bacteria, key gaps in knowledge persist. A key open question in bacteria includes which specific DNA repair pathways mitigate colibactin damage. Given that DNA is fundamentally differently packaged in eukaryotes and bacteria, it remains unknown if colibactin favors A/T-rich regions in bacteria and if it culminates in similar mutations as those reported for colon cells.

Here, we aimed to identify the environmental conditions that maximize colibactin toxicity, and uncover genes that modulate its toxicity using a genome-wide loss-of-function genetic screen. We then investigated if colibactin induces genetic mutations in *E. coli* cells that are cocultured with colibactin-producing cells and if a colibactin-specific mutational signature can be detected. Finally, we evaluated if colibactin production induces self-inflicted damage both in vitro and in silico.

## Results

### Colibactin reduces viability of cocultured *E. coli*

**Figure 1.**
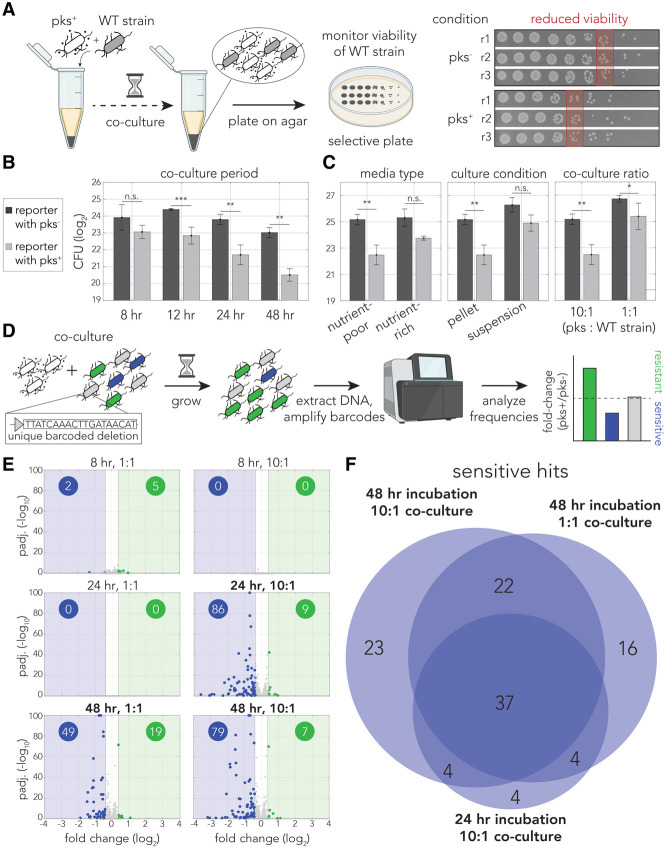
Genetic screen reveals key role of homologous recombination in colibactin response. (*A*) Overview of the reduced viability assay in response to colibactin. Reporter cells were cocultured with pks^+^ cells in a pellet and plated on selective agar to determine the number of viable reporter cells. The colony images on the *right* show representative results of a spotting assay with reduced viability after pks^+^ cocultures. (*B*) Colibactin toxicity correlates with coculture period. A reporter strain was cocultured in pellets with pks^+^ (light gray) or pks^−^ (dark gray) strains at a 10:1 ratio in M9. The bar graphs show the mean CFUs back-calculated from the spotting assay. Error bars show standard deviation of triplicates. (**) *P* < 0.01, (***) *P* < 0.001, (n.s.) not significant, two-sample *t*-test. (*C*) Colibactin toxicity is influenced by coculture conditions. All conditions were evaluated at 24 h. (*Left*) Toxicity is impacted by growth media: Cocultures were conducted in either nutrient-poor (M9) or -rich media (LB) at a 10:1 ratio and pelleted. (*Middle*) Toxicity is impacted by growth conditions: Cocultures were in nutrient-poor media at a 10:1 ratio either pelleted or in suspension. (*Right*) Toxicity is impacted by incubation ratio: Cocultures were in nutrient-poor media at either a 10:1 or 1:1 ratio of pks^+^ to reporter strain and pelleted. Error bars show standard deviation of triplicates. (*) *P* < 0.05, (**) *P* < 0.01, (n.s.) not significant, two-sample *t*-test. (*D*) Overview of barcoded genetic screen approach. (*E*) Volcano plots of screen results. Resistant hits are colored in green, and sensitive hits are colored in blue. The number of hits for each direction are reported in colored circles on each volcano plot. Vertical gray lines represent the fold-change cutoff. (*F*) Venn diagram of shared sensitive hits between 24 h 10:1, 48 h 1:1, and 48 h 10:1 screens.

We first determined if colibactin reduces viability in neighboring bacteria. We cocultured the reporter strain (ampicillin-resistant laboratory strain) with colibactin producers, *E. coli* cells from the same genetic background that harbor the pathogenicity island (pks^+^) on a bacterial artificial chromosome (BAC) (Silpe et al. 2022). We grew cells as a pellet to maximize cell–cell contact for the duration of the coculture experiment. Bacteria transformed with an empty BAC were used as a negative control (pks^−^). Following coculture, we calculated the colony forming units (CFUs) of the reporter strain by plating on selective agar. Figure 1A shows the experimental setup and representative plate images showing reduced viability. We measured viability over 48 h and observed that colibactin reduces viability within 12 h of coculture and continues to decrease viability of cocultured cells over time (Fig. 1B). Because growth of the wild-type strain in the pellet is arrested after the 12 h time point in the control experiment, reduced viability in the pks^+^ condition is likely attributed to cell death and not just growth arrest.

We then wanted to quantify colibactin toxicity under different environmental conditions because previous work showed that colibactin expression is influenced by environmental conditions (Tronnet et al. 2016, 2017; Chagneau et al. 2019; Oliero et al. 2021; Bossuet et al. 2023). We compared cocultures in different media, in pellet or suspension, and at different strain ratios. Figure 1C shows the results of these experiments. We observed that colibactin toxicity was higher in nutrient-poor media compared with nutrient-rich media and was also higher when the coculture grew in a pellet compared with growth in suspension. Lastly, toxicity was increased when the coculture ratio was skewed toward colibactin producers. Taken together, these experiments allowed us to uncover the experimental settings that maximize colibactin toxicity.

### Genetic screen uncovers genes mitigating colibactin toxicity

To identify genes and pathways involved in mitigating colibactin-induced damage, we used a loss-of-function genetic screen that was based on results from our reduced viability assay (Fig. 1C). We used a pooled genetic screening approach that we recently used for studying drug sensitivity (Noto Guillen et al. 2024). Briefly, a collection of 7259 *E. coli* knockout strains targeting 3680 nonessential genes were cocultured with the colibactin-producing strain. Changes in strain frequency were deduced by sequencing unique DNA barcodes that identify each knockout strain. We conducted the screen with different levels of selective pressure (coculture ratios and incubation durations). Cocultures with a pks^−^ strain were used as controls. Overall, we conducted six screens with five biological replicates each (see Methods). At the end of each screen, cells were collected for DNA extraction, barcode amplification, and DNA sequencing (Fig. 1D). In these screens, barcode depletion in the pks^+^ condition relative to the pks^−^ condition is indicative of a gene that mitigates colibactin-induced damage (Fig. 1D).

Analysis of the DNA sequencing results identified at least 2.6 × 10^6^ barcode sequences in each experiment; 95% of individual barcodes were sequenced more than 10 times, and the median depth per barcode was 1161. Figure 1E shows volcano plots for each of the screening conditions. As expected, we detected more hits when the target bacteria had longer exposure to and were in the presence of more colibactin producers (Fig. 1E). We identified a total of 110 knockout strains that conferred colibactin sensitivity and 42 knockout strains that conferred resistance in at least one condition (Supplemental Table S1). When analyzing the overlap in the 110 sensitive hits found across the three most toxic conditions (marked in bold in Fig. 1E), we observed that 67 of them were shared in at least two conditions (Fig. 1F). We noted that contrary to screens we previously performed with this strain collection (Rosener et al. 2020; Sayin et al. 2023), we observed a high degree of variability between biological replicates, manifesting in a marginal *P*-value for some of the top hits (Fig. 1E). We expect that this variability arises from cells randomly detaching from the pellet and growing without consistent colibactin exposure. As detached cells are also collected and used for DNA extraction, they are included in the analysis and can potentially give rise to false-positive hits.

**Figure 2.**
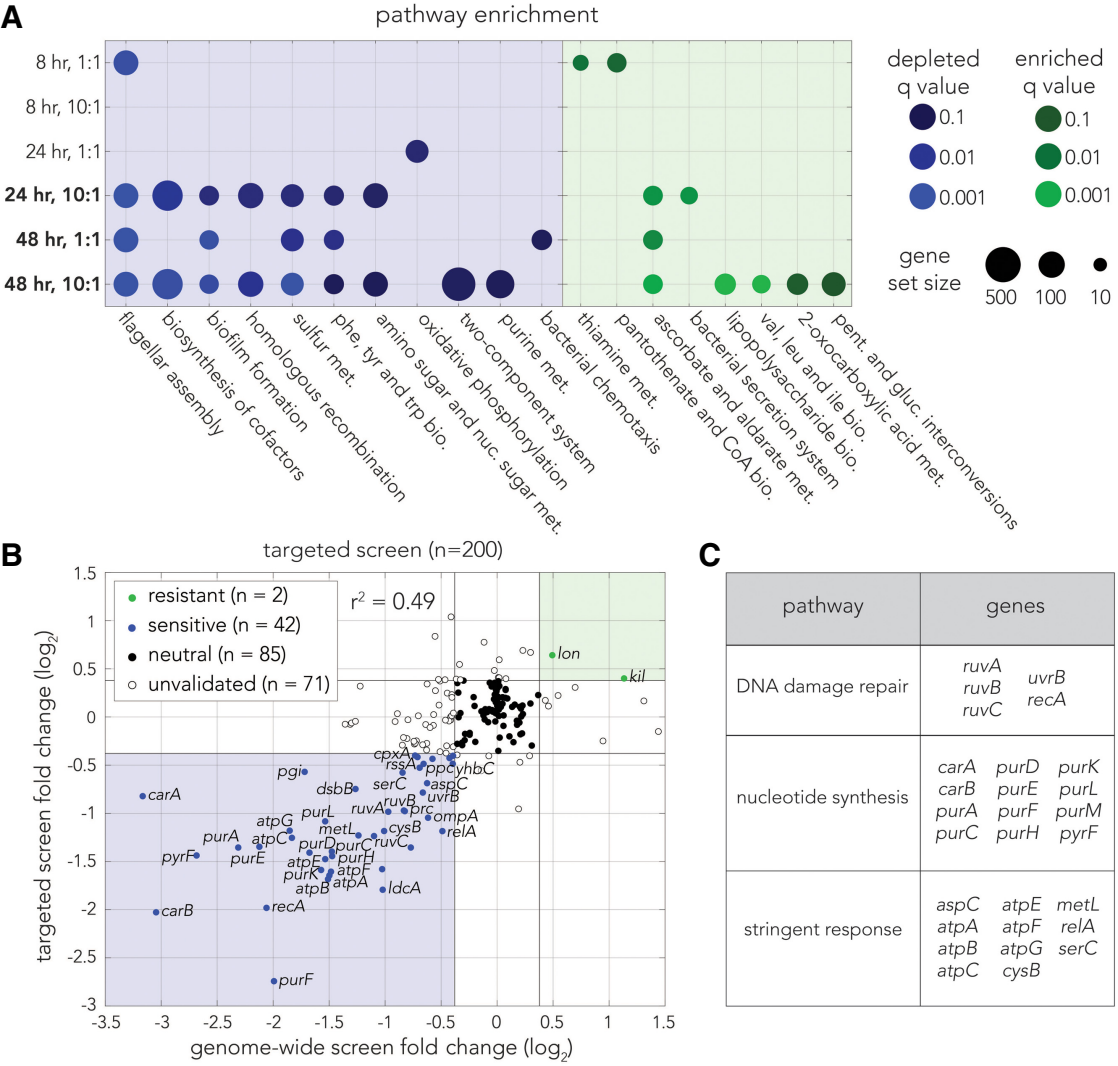
Pathway enrichment and targeted validation screen identify roles of homologous recombination in colibactin response. (*A*) Pathway enrichment analysis using KEGG (Kanehisa and Goto 2000) terms for each screen. Enriched terms are colored in green by *Q*-value, and depleted terms are colored in blue by *Q*-value. Circle size represents the size of the gene set for the respective pathway. (*B*) Comparison of knockout fold-changes in the genome-wide screen and targeted validation screen. Points are colored by consensus enrichment change in the genome-wide screen and targeted screen: green for resistant, blue for sensitive, black for neutral, and empty for unvalidated. Gray lines mark the fold-change cutoff (1.3). (*C*) Key pathways underlying colibactin sensitivity. Twenty-eight of the validated colibactin-sensitive knockouts are associated with three cellular pathways.

We next conducted a functional enrichment analysis of the genome-wide screen using Kyoto Encyclopedia of Genes and Genomes (KEGG) (Kanehisa and Goto 2000) and Gene Ontology (GO) terms (The Gene Ontology Consortium et al. 2000) to evaluate pathways involved in colibactin-induced toxicity (Supplemental Table S2). This analysis identified pathways directly or indirectly connected to DNA synthesis and repair as conferring sensitivity (Fig. 2A). These pathways include homologous recombination, purine metabolism, and the master pathway for amino sugar and nucleotide sugar metabolism, which is connected to the stringent response. Knockout of the flagellar assembly pathway also increased sensitivity, which may potentially be owing to the inability of these strains to escape the coculture pellet.

Given the relatively high variability we observed between replicates in the genome-wide screen, we followed with a targeted validation screen. This screen included all 162 original hits and 38 additional neutral knockout strains. We performed the screen with six replicates at the most extreme coculture condition (colibactin:target bacteria at a 10:1 ratio for 48 h). Figure 2B shows a comparison between the genome-wide and targeted screens (*r*^2^ = 0.49, *P*-value < 10^−10^ by Pearson's correlation test). Results for all 200 strains in the validation screen appear in Supplemental Table S3. Inspection of the genes mitigating colibactin-induced damage revealed hits from three central pathways involved in DNA damage repair (Fig. 2C). We found multiple genes associated with homologous recombination (*ruvABC* and *recA*) (Kowalczykowski et al. 1994) but only one from the nucleotide excision repair pathway (*uvrB*), which was previously linked to self-inflicted colibactin toxicity (Bossuet-Greif et al. 2016). We noted that knockouts from other DNA repair pathways were also observed to increase colibactin sensitivity, yet they were below our cutoff value that we chose for fold-change. These knockouts included *uvrA* of the nucleotide excision repair pathway and *umuCD* that encodes polymerase V involved in translesion synthesis (Supplemental Table S1). We also found 12 sensitive hits from the nucleotide synthesis gene network, likely increasing sensitivity to DNA damage by reducing nucleotide availability (Rosener et al. 2020). Lastly, 11 of the sensitive hits belong to the stringent response that works along with the SOS response to induce mutagenic DNA repair (Ponder et al. 2005; Shee et al. 2011; Zhai et al. 2023).

Taken together, our screens revealed that inactivation of multiple pathways increases colibactin sensitivity and that they all likely operate by modulating the DNA damage response. Moreover, within the DNA damage response, we primarily detected genes involved in homologous recombination but only one gene linked to nucleotide excision repair.

### Colibactin induces a specific mutational signature

**Figure 3.**
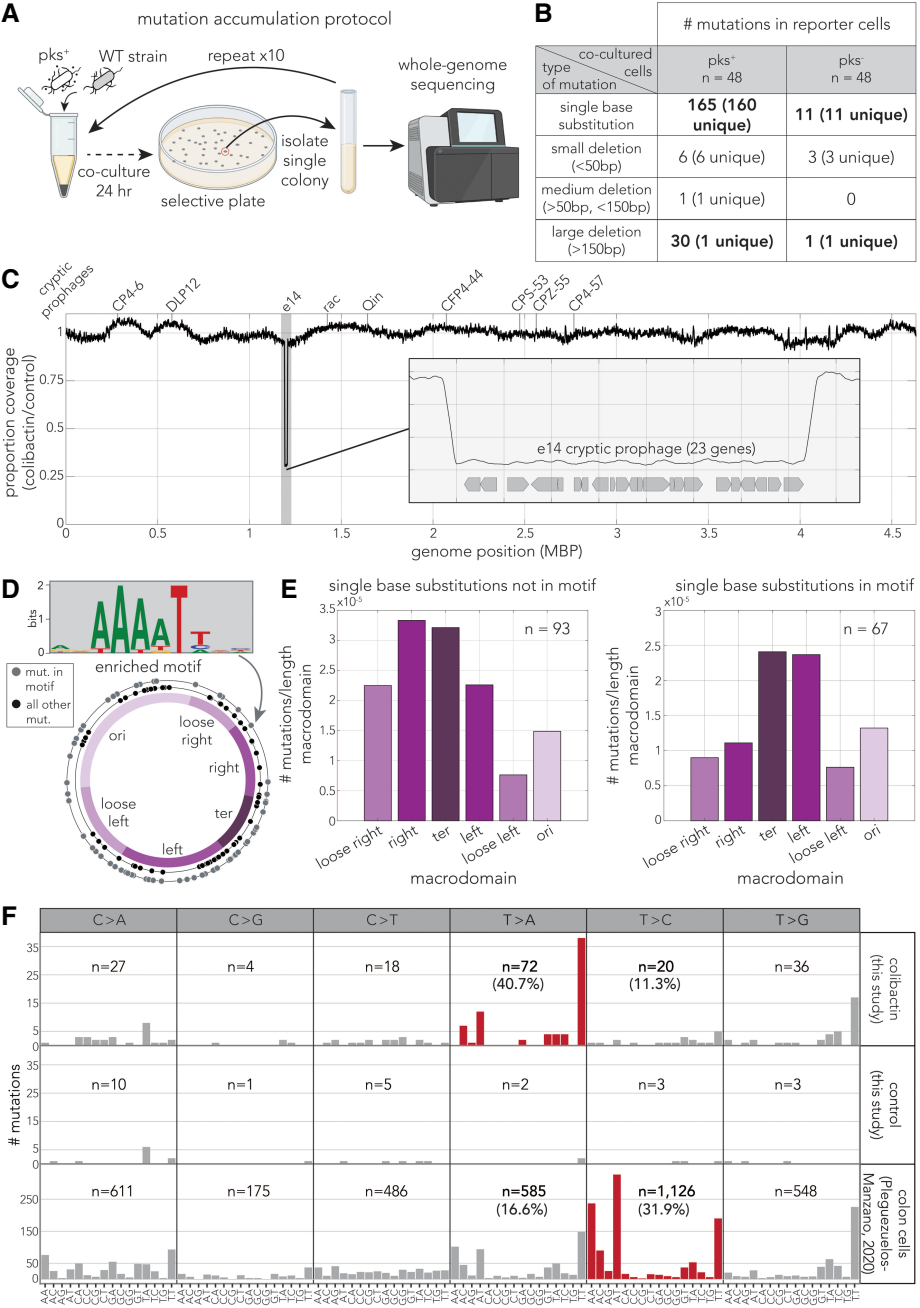
Colibactin induces a bacteria-specific mutational signature. (*A*) Overview of mutation accumulation experiment. Reporter cells were cocultured with pks^+^ cells in a pellet for 24 h before plating on selective agar. Single reporter colonies were selected and grown for subsequent exposure. Reporter cells were exposed 10 times before whole-genome sequencing. (*B*) Summary table of annotated mutations by mutation type. Bold mutations mark key differences between the pks^+^ and pks^−^ exposed populations. (*C*) Whole-genome sequencing coverage of colibactin/control conditions. The positions for all nine cryptic prophages are marked *above*. The gray-shaded region marks the e14 prophage region shown in more detail in the panel *inset*. (*D*) The A/T enriched motif found in the 13 bp region that surrounds positions of single-base substitutions (SBSs). The ring plots show positions of SBSs: outer ring in gray indicates SBSs matching the motif; inner ring in black, all other SBSs. The chromosome ring shows previously defined macrodomain regions. (*E*) SBS positions show genomic positional bias and are enriched near the terminus, increasingly so for mutations occurring in the identified motif. (*F*) Trinucleotide context of SBS mutations. The *upper* panel shows mutations identified after coculturing with pks^+^ bacteria. The *middle* panel shows mutations identified after coculturing with pks^−^ bacteria (control). The *bottom* panel shows mutations annotated in colon cells exposed to pks^+^ bacteria by Pleguezuelos-Manzano et al. (2020). Differential mutation signatures between our work and work in colon cells are highlighted in red.

Colibactin-induced damage leads to a specific mutational signature in colorectal cancers (Dziubańska-Kusibab et al. 2020; Pleguezuelos-Manzano et al. 2020; Chen et al. 2023) that is attributed to its favorable binding to the particularly narrow minor groove in A/T-rich sequence motifs (Dziubańska-Kusibab et al. 2020). We sought to determine whether the mutational pattern is the same in bacteria using mutation accumulation experiments with repeated exposure to colibactin producers. Ampicillin-resistant DNA damage reporter cells were cocultured for 24 h with genetically engineered colibactin producers and spotted on selective agar. A single reporter colony was then grown and used for a subsequent exposure cycle. We repeated the experiment with 48 independent replicates exposed to colibactin (pks^+^) and 48 controls (pks^−^). After completing 10 exposure cycles, we sequenced the genome and annotated the mutations with the breseq tool (Fig. 3A; Deatherage and Barrick 2014). Because colonies were selected randomly in each cycle, observed mutations are not expected to be adaptive ones but should reflect the pattern of spontaneous mutation induced by colibactin exposure.

Figure 3B shows a summary of the observed mutations by category, which are also reported in Supplemental Table S4. Overall, we observed considerably more mutations across all categories in the pks^+^ coculture condition. We observed a 10-fold increase in single-base substitutions (SBSs) and only a twofold increase in short indels from coculture with pks^+^ cells (short indels are highly prevalent in colon cells) (Pleguezuelos-Manzano et al. 2020; Chen et al. 2023). To account for transcription coupled repair, we also tested for mutational strand bias. We did not detect a SBS bias when comparing the transcribed and untranscribed strands. Lastly, we also observed a very high frequency of a single large deletion that was almost identical across 30 of the 48 pks^+^ coculture replicates. The genome position of this deletion aligns with the known location of the cryptic prophage e14 (Fig. 3C) and agrees with the known role of colibactin in inducing prophage excision (Silpe et al. 2022). A high frequency of e14 excision relative to other cryptic prophages was also observed after exposure to the mitomycin-C alkylating agent, which also causes DNA damage (Wang et al. 2010).

We analyzed the genomic context surrounding SBSs to check whether specific motifs are enriched for colibactin-induced mutations using the STREME (Bailey 2021) tool. Figure 3D shows the statistically significant motif (*P*-value = 0.003) that was found in 67 of 160 unique SBS. The A/T-rich motif we found was similar to the motif identified in colorectal cancers (Dziubańska-Kusibab et al. 2020; Pleguezuelos-Manzano et al. 2020). Within this motif, the mutated base was either the A in position 4 or the T in position 7. We next tested if enriched SBS positions show a spatial preference using previously annotated chromosome macrodomains (Lioy et al. 2018), as shown in Figure 3D. We observed that all SBS sites, those matching the motif and those not matching it, were commonly closer to the terminus region (Fig. 3D,E, ter). Finally, we directly compared the mutation patterns we found with those reported for colon organoids (Pleguezuelos-Manzano et al. 2020) by looking at the trinucleotide context of the mutated base (Fig. 3F). This comparison showed that despite similarity in the genomic context, a striking difference exists in the mutation outcome. As Figure 3F shows, mutations in A/T-rich trinucleotides in *E. coli* were predominantly T > A, whereas in colon cells they were predominantly T > C.

In summary, we found similarities, but also clear differences, between colibactin-induced mutations in bacteria and colon cells. In both cases, increased mutation rates are clearly detected and show a bias toward A/T-rich sequence motifs. However, differences exist in the nucleotide that results from the mutation event and far-reduced frequency of indels in *E. coli* relative to colon cells. Lastly, we detected that colibactin-induced mutations were biased in some genome macrodomains in *E. coli*.

### Self-inflicted damage in colibactin-producing *E. coli*

**Figure 4.**
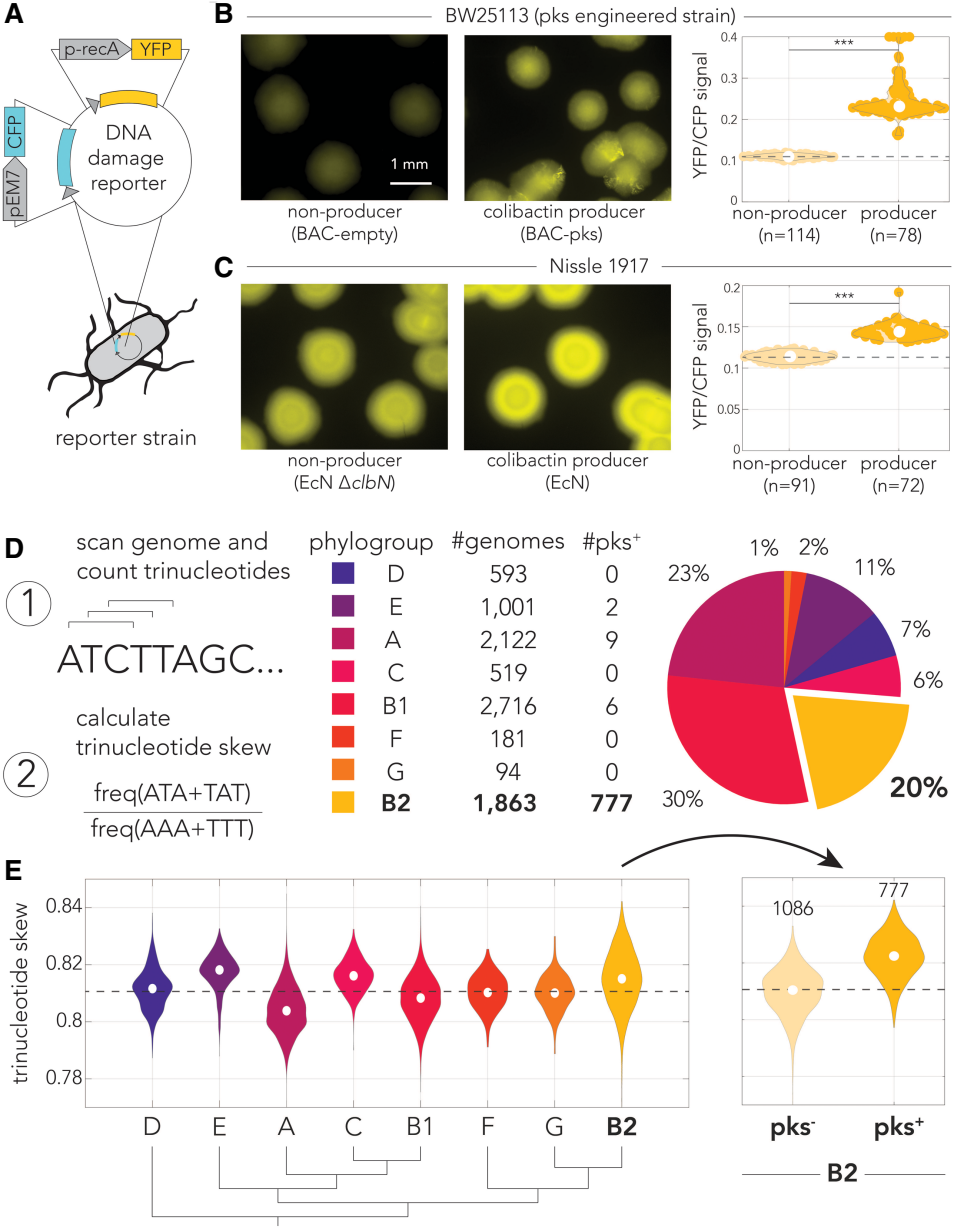
Colibactin inflicts self-damage. (*A*) Schematic of the fluorescent DNA damage reporter plasmid used to quantify self-damage. CFP is expressed under a constitutive promoter, and YFP is expressed under a DNA damage–inducible promoter. Representative microscopy images of the engineered (BAC) pks^+^ and pks^−^ colonies (*B*) or Nissle 1917 pks^+^ (EcN) and pks^−^ (EcN Δ*clbN*) expressing our *recA* reporter plasmid (*C*). Images show YFP expression, which represents *recA* activation in the colonies owing to colibactin-induced self-damage. The fluorescence intensity range was set according to the intensity observed in the colibactin-producer field of view per strains. Violin plots display the median YFP signal intensity per colony for each strain. Background YFP and CFP autofluorescence of the colonies was subtracted from each channel before YFP was normalized to CFP per colony (***) *P* < 0.001, two-sample *t*-test. (*D*) Schematic of our analysis of 9089 genomes to calculate the trinucleotide skew toward ATA/TAT over AAA/TTT sequences. Genomes are broken down by phylogroup, and the total number of genomes per phylogroup, as well as the number of pks^+^ genomes, included in our analysis is reported. The pie chart shows the total percentage of genomes each phylogroup represented. (*E*, *left*) Violin plots of trinucleotide skewness by *E. coli* phylogroup. Median skew is marked by the dashed line. (*Right*) The B2 phylogroup skewness is further divided into genomes with colibactin and genomes without colibactin. The number of genomes in each condition is labeled in this panel.

Colibactin self-protection is attributed to two mechanisms: colibactin activation by the ClbP peptidase after it is exported to the periplasm (Dubois et al. 2011; Brotherton and Balskus 2013; Velilla et al. 2023) and inactivation of intracellular colibactin by the ClbS cyclopropane hydrolase (Bossuet-Greif et al. 2016; Tripathi et al. 2017). Given that we did not identify genes involved in colibactin import in our genetic screen, we hypothesized that mature colibactin may permeate back into producing cells. We therefore further hypothesized that producer cells might experience weak, yet elevated levels, of DNA damage despite self-protection mechanisms. We first tested this hypothesis by introducing a fluorescent DNA damage reporter into the pks^+^ and pks^−^ strains (Supplemental Methods). We constructed the fluorescent DNA damage reporter by cloning a YFP fused to the *recA* gene promoter, known to respond to DNA damage (Vollmer et al. 1997), and a CFP under control of a constitutive promoter into a low-copy plasmid (Fig. 4A). The CFP tag allowed us to identify reporter cell colonies and normalize DNA damage–induced fluorescence (YFP) to cell numbers in reporter colonies.

These strains were then grown on agar to quantify the intensity of the fluorescent reporter. Figure 4B shows representative microscopy images of colibactin producers and nonproducers. In agreement with our hypothesis, we indeed observed increased DNA damage reporter activity in cloned cells harboring the *pks* pathogenicity island relative to control nonproducers. Quantification of the median reporter level across dozens of colonies revealed this increase is statistically significant (Fig. 4B, right). Because the colibactin expression level in genetically engineered clones may be higher than expression levels of strains naturally harboring the *pks* pathogenicity island, we repeated this experiment with the Nissle 1917 strain that naturally expresses colibactin (Fig. 4C). We again observed elevated reporter activity in colibactin producers relative to nonproducing *clbN* knockouts (Fig. 4C, right). As expected, this increase was relatively weak compared with the engineered pks^+^ strain in Figure 4A as well as a *clbS* knockout that is unable to inactivate colibactin to protect the producing cells (Supplemental Fig. S1).

Motivated by the observation that colibactin producers show increased DNA damage reporter activity, we hypothesized that self-inflicted damage may also be evident in the genome of strains harboring the *pks* pathogenicity island. We reasoned that this prediction can be evaluated by examining whether there is a bias in trinucleotide sequences in genomic DNA. Specifically, we predicted colibactin-induced DNA damage will be associated with a bias toward ATA and TAT trinucleotides at the expense of AAA and TTT trinucleotides (Fig. 3F). In a random genome, we would expect equal proportions of these trinucleotides; thus, shifts in these proportions can be measured as an indicator of colibactin-induced DNA damage. We validated this expectation in a mock random genome using a sliding window of 3 nucleotides to calculate the proportion of trinucleotides matching ATA/TAT or AAA/TTT. This simulation yielded equal proportions of each complementary trinucleotide sequence, suggesting that using a sliding window does not bias our results.

We tested our hypothesis in 9089 annotated genomes spanning all *E. coli* phylogroups (Fig. 4D) and calculated a trinucleotide skewness metric (N_ATA_ + N_TAT_)/(N_AAA_ + N_TTT_) to detect any bias toward colibactin-associated trinucleotide sequences. This metric is not strand-specific as we pooled together complementary trinucleotides in this calculation (e.g., N_ATA_ + N_TAT_). The strains included in this analysis and their calculated trinucleotide skew are reported in Supplemental Table S5. Of the 9089 genomes analyzed, 794 were annotated as containing the *pks* island, with the majority (777) belonging to the B2 phylogroup. The almost exclusive occurrence of pks^+^ strains in the B2 phylogroup agrees with previous findings (Nougayrède et al. 2006; Wami et al. 2021). Figure 4E shows the skewness levels calculated for all genomes classified by phylogroup. Calculating the skewness for each phylogroup allowed for setting an expectation on the amount of skew in a phylogroup without the pks island. In agreement with our prediction, we found that the B2 phylogroup, as the most prevalent phylogroup containing the *pks* island (Nougayrède et al. 2006; Wami et al. 2021), had one of the largest ranges of skew with the trinucleotide skew metric. Moreover, separating the strains of this phylogroup to pks^+^ and pks^−^ subgroups (Fig. 4D, right panel) revealed that the skewness is significantly increased specifically in pks^+^ strains (*P*-value < 10^−10^). The range of the skewness in the separated pks^+^ strains and pks^−^ strains in the B2 phylogroup is similar to the other *E. coli* phylogroups. Taken together, our microscopy and genomic analysis results indicate that colibactin-producing strains likely experience elevated levels of basal DNA damage culminating in noticeable bias in trinucleotide genomic composition that is compatible with colibactin self-inflicted damage.

## Discussion

Colibactin-producing bacteria are not uncommon in the gut microbiome of healthy humans, yet their increased prevalence is evident in multiple human diseases ranging from inflammatory bowel disease to colon cancers (Buc et al. 2013; Eklöf et al. 2017; Dejea et al. 2018; Iyadorai et al. 2020). Compelling evidence from colorectal tumors in humans strongly supports the premise that colibactin acts as a tumorigenic mutagen (Dziubańska-Kusibab et al. 2020; Pleguezuelos-Manzano et al. 2020). The clinical relevance of colibactin-induced damage has motivated intense research into the mechanisms underlying its toxicity in eukaryotes, but left fundamental questions underexplored in bacteria. Our study addressed some of these gaps in knowledge and revealed differences between the bacterial and mammalian cellular response to colibactin.

Our study of the cellular mechanisms underlying colibactin damage relied on a loss-of-function genetic screen. This genome-wide approach, applied for the first time to study colibactin toxicity, was unbiased by current understandings of colibactin mode of action. We identified various DNA damage response pathways whose inactivation increased colibactin sensitivity, uncovering a crucial role for homologous recombination (Fig. 2A,C). We only detected a single hit from the nucleotide excision repair pathway, despite its known role in removing interstrand cross-links (Cole 1973; Bossuet-Greif et al. 2016). The findings from our genome-wide genetic screen strongly agree with the current understanding that colibactin-induced toxicity is owing to its role as a DNA-damaging agent. This DNA damage likely underlies the frequent excision of prophages that we (Fig. 3C) and others observed (Silpe et al. 2022).

A novel finding of our work emerged from mutation accumulation experiments. We found that in bacteria, similar to eukaryotes (Dziubańska-Kusibab et al. 2020; Pleguezuelos-Manzano et al. 2020), SBS mutations were primarily located in A/T-rich DNA sequences (Fig. 3D,F). This similarity can be rationalized by the structural model suggesting that colibactin targets these A/T-rich sequences owing to their particularly narrow minor groove (Dziubańska-Kusibab et al. 2020). Despite a similar binding preference, colibactin-induced mutations were markedly different: In colon cells, T > C was the predominant change, whereas T > A was most prevalent in bacteria (Fig. 3F). The difference in DNA repair mechanisms between bacteria and mammalian cells is one plausible mechanism that may underly this mutational dissimilarity. Additionally, indel mutations are abundant in colon cells (Pleguezuelos-Manzano et al. 2020) but uncommon in bacteria. We also detected a positional bias in colibactin-induced mutations in bacteria (near the terminus).

Our genetic screen uncovered that homologous recombination, an error-free repair pathway, plays a major role in mitigating colibactin-induced DNA damage. However, results from our mutation accumulation experiments revealed that colibactin is mutagenic in *E. coli*. This seeming inconsistency may be explained by the complexity of the DNA damage response colibactin induces in *E. coli*, which combines both error-prone and error-free repair mechanisms. Following activation of the SOS response, nucleotide excision repair, an error-free mechanism, repairs damage, but extensive damage can activate polymerase V involved in translesion synthesis, which is error-prone (Maslowska et al. 2019). Our screen results suggested that all these pathways (homologous recombination, nucleotide excision repair, and translesion synthesis) indeed participate in mitigating colibactin-induced damage. It is also intriguing to note that colibactin induces a specific SBS profile. Different SBS patterns were previously reported for other DNA-damaging agents such as UV and mitomycin-C (Kowalczykowski et al. 1994; Maslowska et al. 2019). The divergent mutation patterns reported for these different agents likely arise from a combination of diverse responses to the types of damage and the different DNA sequences being impacted by each of the agents.

Finally, we leveraged the bacteria-specific mutational bias we discovered to investigate if colibactin inflicts self-damage in producers. We found that in both engineered and naturally producing cells, colibactin inflicts self-toxicity that was visualized and quantified with a fluorescent DNA damage reporter (Fig. 4B,C). This is likely because of small quantities of the toxin re-entering the producing cells and binding DNA before it is inactivated by the ClbS cyclopropane hydrolase. A comparative analysis of almost 2000 *E. coli* genomes from the B2 phylogroup provides supporting evidence of colibactin-linked skewness in trinucleotide composition in strains harboring the *pks* pathogenicity island. The skewness in pks^+^ strains of the B2 phylogroup was highest among all phylogroups of the *E. coli* species. Hence, it seems that colibactin entails a cost on producing cells and leaves an evolutionary footprint in their genomes.

Taken together, our systematic work reveals unique features of colibactin toxicity in bacteria. The focus of this study on the effects of colibactin in bacteria is important given that this toxin is commonly found in nonpathogenic strains and therefore likely emerged, like many other bacterial toxins, to facilitate competition within microbial communities (Kern et al. 2021). Our study outlines an important direction for future investigation. This key direction builds on the newly identified bacteria-specific mutational signature. Specifically, it will be interesting to explore if evidence of colibactin-specific signatures can be found in longitudinal microbiome samples from individuals that harbor colibactin-producing bacteria in their gut. Quantifying the strength and rate of a colibactin-linked mutational bias may provide a noninvasive method to estimate the intensity of colibactin exposure in a specific individual. Given that 30% of healthy individuals harbor pks^+^ strains in their gut microbiome, additional information about the rate of colibactin damage accumulation in the individual's microbiome may help to gauge their risk for developing colibactin-linked colon cancer.

## Methods

### Media and growth conditions

All strains used in this study are reported in Table 1. All experiments were performed in either luria broth (LB) or minimal synthetic media (M9 salts supplemented with 0.4% glucose, 2 mM MgSO_4_, 0.1 mM CaCl_2_, 0.2% Amicase). Overnight cultures for all experiments were grown at 37°C with 200 rpm orbital shaking. During the overnight growth of antibiotic-resistant strains, we added antibiotics at the following concentrations: 50 μg/mL spectinomycin, 50 μg/mL kanamycin, 25 μg/mL chloramphenicol, and 50 μg/mL carbenicillin.

**Table 1.** Bacteria strains used in this study

Strain	Nickname	Source	Use
BW25113 Δ*gspI*::carb	Viability reporter strain; ampicillin-resistant reporter strain	This study	Colibactin impact on cell viability, mutation accumulation
BW25113 pBeloBAC11 + pks	Engineered pks^+^	(Silpe et al. 2022)	Colibactin impact on cell viability, genetic screens
BW25113 pBeloBAC11	Engineered pks^−^	(Silpe et al. 2022)	Colibactin impact on cell viability, genetic screens
Pooled *E. coli* library	Pooled *E. coli* library	Hirotada Mori, Nara Institute of Science and Technology, Japan	Genetic screens
BW25113 pBeloBAC11 + pks pRecA	Engineered pks^+^ with DNA damage reporter	This study	Self-inflicted damage
BW25113 pBeloBAC11 pRecA	Engineered pks^−^ with DNA damage reporter	This study	Self-inflicted damage
Nissle 1917 pRecA	Nissle 1917 pks^+^ DNA damage reporter	This study	Self-inflicted damage
Nissle 1917 Δ*clbN*::chl pRecA	Nissle 1917 pks^−^ DNA damage reporter	This study	Self-inflicted damage
Nissle 1917 Δ*clbS*::chl pRecA	Nissle 1917 pks^+^ *clbS* knockout DNA damage reporter	This study	Self-inflicted damage

### Cloning deletion strains

gspI was deleted from BW25113 using lambda red recombination with an insert containing 60 bp homology arms to the upstream and downstream genomic regions and a carbenicillin-resistance cassette. clbN and clbS were deleted from Nissle 1917 using lambda red recombination with an insert containing 40 or 60 bp homology arms to the upstream and downstream genomic regions, respectively, and a chloramphenicol-resistance cassette (Supplemental Methods).

### Monitoring colibactin impact on viability of cocultured cells

Cultures of the viability reporter strain and the engineered pks^+^ and pks^−^ strains were grown overnight in LB. One milliliter of overnight culture was washed three times with PBS. Cultures were then diluted 1:50 into either LB or M9 and grown for 2 h. Following growth, OD_600_ was measured, and cultures were diluted to OD_600_ = 0.1. Reporters and engineered pks^+^ and pks^−^ strains were mixed in 96-deep-well plates (Eppendorf 2231000920) to a final volume of 500 μL at a 10:1 or 1:1 ratio (producers to reporters). For the pelleted coculture conditions, plates were centrifuged at 4000*g* for 6 min and incubated at 37°C with no shaking. For suspension coculture conditions, plates were incubated at 37°C with 200 rpm orbital shaking.

At each time point, cocultures were thoroughly mixed by pipetting them, and 5 μL aliquots were transferred to the top row of a 96-well microplate (FisherBrand FB012932) containing 95 μL PBS. Samples were serially diluted 1:5 over the seven remaining rows in the microplate. From each dilution well, 4 μL was spotted on LB agar plates supplemented with spectinomycin to select for the reporter strain. We then back-calculated CFUs in each sample based on the dilution factor of the least dilute spot for each sample that contained three to 25 colonies.

### Genome-wide loss-of-function genetic screen

We used a pooled genetic screening approach that we previously developed (Rosener et al. 2020; Noto Guillen et al. 2021, 2024; Sayin et al. 2023) to identify genes and pathways impacting colibactin sensitivity. The method relies on a collection of 7259 knockout strains that span 3680 nonessential genes in *E. coli*. Each knockout strain harbors a 20 bp nucleotide barcode that is integrated into its chromosome. This collection allows for performing pooled genetic screens and identifying the frequency of each knockout strain by targeted deep sequencing of the barcode locus.

A 200 μL aliquot of frozen glycerol stocks of the pooled *E. coli* library was grown overnight in LB supplemented with chloramphenicol. The engineered pks^+^ and pks^−^ strains were also grown overnight in LB supplemented with chloramphenicol. Cultures were washed twice in PBS and resuspended in M9. The knockout collection was diluted 1:50 in 40 mL M9. The engineered pks^+^ and pks^−^ strains were diluted 1:50 in 100 mL M9. Cultures were grown for two additional hours at 37°C and 200 rpm to allow for adjustment to media and exit from stationary phase before adjusting their density to OD_600_ = 0.1 in 60 mL for the knockout collection and 140 mL for the engineered strains. The cultures were mixed at a 1:1 and 10:1 ratio and then divided into 8 mL replicates spread across 16 wells in a 96-deep-well plate (Eppendorf 2231000920). Cocultures were pelleted at 4000*g* for 6 min before being incubated for 8, 24, and 48 h at 37°C . Cultures were then resuspended, and all wells per replicate were merged into a conical 50 mL tube. The cultures were pelleted at 4000*g* for 6 min, and media were aspirated. Pellets were flash-frozen and stored at −80°C until DNA was extracted using a Zymo Quick-DNA midiprep plus kit (D4075).

DNA concentrations were measured with Quant-iT dsDNA high-sensitivity assay (Invitrogen Q33232) on a Tecan Spark plate reader, and samples were normalized to 20 ng/μL. The barcode sequencing protocols we previously developed were modified to account for the low proportion of DNA in each sample that originated from the knockout library: DNA template for each PCR reaction was increased to 20 ng, and four reactions were set up per sample to get sufficient coverage of the library (based on calculations of the final CFUs of reporter cells and pks^+^ cells in spotting cocultures). The template DNA was amplified over 23 cycles with custom forward and reverse primers and 2× KAPA HiFi HotStart ReadyMix (Kapa Biosystems KK2602). Following barcode amplification, the four reactions were pooled, and 25 μL was purified for downstream use. The PCR product was purified with AMPure XP beads (Beckman Coulter A63881) following the standard protocol of beads added at a 0.9 × sample volume. We modified the Nextera XT index kit (Illumina FC-131-1024) protocol to work with half volumes. These products were then run on a 2.5% agarose gel and extracted using a ZR-96 Zymoclean gel recovery kit (D4021). The purified libraries were quantified with the Quant-iT dsDNA high-sensitivity assay and normalized to 4 nM. Library quality was assessed on a Bioanalyzer with the Agilent high-sensitivity DNA kit (Agilent Technologies 5067-4626). Libraries were denatured and diluted according to the NextSeq 500/550 system protocol and sequenced with the NextSeq 500/550 high-output kit v2.5, 75 cycles (Illumina 20024906) on a NextSeq 500/550 machine.

### Analysis of genetic screen results

We extracted barcode counts from FASTQ sequencing files using a custom MATLAB (MathWorks) script. Exact matches of barcodes (15–25 bp) were searched for in each read, and a knockout strain was assigned if there was a matching barcode. Any nucleotide with a quality score below 10 was masked in the analysis. Counts of knockout strains targeting the same gene were summed together. Knockouts that impacted sensitivity to colibactin were identified by comparing the relative frequency of a knockout strain in the pks^+^ coculture condition with the relative frequency in the pks^−^ coculture condition. The statistical significance of the changes in relative frequency was determined with the DESeq2 tool (Love et al. 2014). We chose log_2_-fold-change (>1.3) and adjusted *P*-value thresholds (<0.25) to classify resistant and sensitive knockouts. We used the gene set enrichment analysis tool GAGE (Luo et al. 2009) to test for functional enrichment. For this analysis we used the KEGG (Kanehisa and Goto 2000) and GO (The Gene Ontology Consortium et al. 2000) databases.

### Targeted validation genetic screen

Knockout strains for all resistant and sensitive hits determined by the genome-wide genetic screen (including several marginally sensitive [four] or resistant [five] strains) along with 38 neutral strains were picked from glycerol stocks of single-knockout strains composing the genome-wide library. Strains were cultured in 1 mL LB supplemented with chloramphenicol overnight. The engineered pks^+^ and pks^−^ strains were also grown overnight in LB supplemented with chloramphenicol. The following day, 100 μL of each resistant or sensitive knockout strain culture was combined along with 400 μL of each neutral knockout strain to pool all strains together to a single culture. From the combined strains, 2 mL was taken to wash, along with 2 mL of each engineered overnight culture. Cultures were washed twice in PBS before resuspending in M9 media. Both engineered strains were diluted 1:50 in 100 mL of M9. The pooled knockout strains were diluted 1:50 in 45 mL of M9. The diluted cultures were incubated and grown for 3 h to adjust to the M9 media and exit stationary phase. Then the culture density was measured with OD_600_, and cultures were diluted to OD_600_ = 0.1. The cultures were mixed in 10:1 (produces to reporters) ratios and divided into 8 mL replicates spread across 16 wells in a 96-deep-well plate (Eppendorf 2231000920). Cocultures were pelleted at 4000*g* for 6 min before being incubating for 48 h (after 24 h, cultures were mixed and repelleted) at 37°C. Cultures were then resuspended, and all wells per replicate were merged into a conical 50 mL tube. The cultures were pelleted at 4000*g* for 6 min, and media were aspirated. Pellets were flash-frozen and stored at −80°C until DNA was extracted using Zymo Quick-DNA midiprep plus kit (D4075). DNA Library preparation and targeted sequencing were identical to the genome-wide screen.

### Mutation accumulation

An ampicillin-resistant BW25113 strain and the engineered pks^+^ and pks^−^ strains were grown overnight on LB supplemented with antibiotics. One milliliter aliquots of each overnight culture were washed in PBS three times before 1:50 dilution in M9 followed by growth for 2 h at 37°C. Cultures were then normalized to OD_600_ = 0.1. Reporters and engineered pks^+^ and pks^−^ strains were mixed in 96-deep-well plates (Eppendorf 2231000920) to a final volume of 500 μL at a 10:1 ratio (producers to reporters) and pelleted by centrifuge at 4000*g* for 6 min. The cultures were incubated without shaking for 24 h at 37°C . Wells were then mixed, and 5 μL of each coculture was transferred to a 96-well microplate (FisherBrand FB012932) and diluted by mixing with 95 μL of PBS. From there, each culture was serially diluted 1:5 for seven dilutions total. Four microliters from each dilution was spotted on LB agar plates supplemented with carbenicillin to select for the ampicillin-resistant BW25113 strain. The following day, a single colony was picked from each replicate from the spotting agar plate and inoculated into 500 μL M9 in a 96-deep-well plate. Fresh cultures of the engineered pks^+^ and pks^−^ strains were grown overnight, and the coculture protocol was repeated. This process, from diluting the 24 h cocultures, spotting, expanding single colonies, and setting up new cocultures, was repeated for 10 cycles. After that, single colonies were picked from each replicate, expanded, and frozen as glycerol stocks.

Glycerol stocks were inoculated into 1 mL LB and grown for 3–4 h before DNA was extracted with the Zymo Quick-DNA 96 kit (D3012) and quantified with a Quant-iT dsDNA high-sensitivity assay (Invitrogen Q33232). Samples were normalized to 12 ng/μL. Prior to sequencing, four replicates from each coculture condition were pooled evenly by combining 10 μL of each 12 ng/μL stock. The pooled samples (24 total) were sent for whole-genome sequencing at SeqCenter (Illumina paired-end sequencing with 2 × 151 bp). The average coverage per genome was 65. Reads were aligned to the reference genome (obtained from the NCBI GenBank database [https://www.ncbi.nlm.nih.gov/genbank/] under accession number CP009273) with breseq (Deatherage and Barrick 2014) to identify mutations. The tool was used in population mode (we discarded mutations with a proportion below 0.1). Mutations occurring in both the pks^+^ and pks^−^ conditions were excluded as they likely existed in the ancestor. To annotate large deletions, we determined genome coverage of each sample with a custom Python script using a sliding window of 10,000 bp. We examined the coverage by plotting it after further smoothing in MATLAB (MathWorks). Sequence encompassing 6 bp upstream of and downstream from single-base substitutions was analyzed for enriched motifs using STREME (Bailey 2021). Because only a few mutations were identified in the pks^−^ condition, we selected 1000 random 13 bp sequences from the reference genome to use as a control. Sequences containing the enriched motif were identified using FIMO (Grant et al. 2011) with the probability matrices generated by STREME. The frequency of the enriched motif sequences was plotted across previously defined macrodomains in the *E. coli* genome (Lioy et al. 2018).

### DNA damage fluorescent reporter

We cloned the DNA damage reporter plasmid with the Gibson assembly method (Gibson et al. 2009) using In-Fusion snap assembly master mix (Takara 638947) (Supplemental Methods). In a single assembly reaction, we integrated a YFP and the *recA* promoter into a plasmid backbone containing a spectinomycin-resistance cassette and CFP. When amplifying the backbone, we also replaced the CFP promoter with a 48 bp EM7 promoter that was encoded on the amplification primer. The 81 bp *recA* promoter was amplified from the BW25113 genome (the promoter region was defined according to previous work) (Pagès et al. 2003; Stohl et al. 2003; Salgado et al. 2024). The final plasmid was a low-copy plasmid with the sc101 origin and spectinomycin resistance. The plasmid assembly was validated with Sanger sequencing spanning the integration sites.

### Self-inflicted damage microscopy

The engineered pks^+^ and pks^−^ strains and the Nissle strains (pks^+^, pks^−^, and pks^+^ *clbS* knockout) harboring the DNA damage reporter were grown overnight in LB supplemented with antibiotics. OD_600_ was measured, and each culture was subsequently diluted so that plating on agar plates would yield about 200 colonies. The diluted cultures were spread with glass beads on M9 agar plates and were incubated at 37°C. We imaged 40–70 fields of view for each plate with a Zeiss Axio Observer.Z1 epifluorescence microscope. Colonies were imaged at 2.5 × magnification using CFP (475 nm), YFP (524 nm), and brightfield channels. Analysis was performed using custom MATLAB scripts (MathWorks). Briefly, colonies were segmented using an automatically determined threshold on the CFP channel image. To ensure that we only analyzed whole, single colonies, we excluded masked regions contacting the image border and filtered masks by area and circularity. Within each mask, the median YFP signal was measured. Background autofluorescence of untagged colonies was subtracted from both YFP and CFP before the YFP signal of each colony was normalized to its CFP signal.

### Bioinformatics analysis of colibactin-linked trinucleotide skew

We tested the trinucleotide composition of 9089 *E. coli* genomes downloaded from the NCBI genome database that were previously assigned to a specific phylogroup (Abram et al. 2021). We discarded genome assemblies that had genome lengths below 4 Mb and those that were flagged as “status: suppressed” by RefSeq annotation (this flag points to potential concerns with the genome assembly). For each genome, we scanned the DNA sequences of all contigs and calculated the frequencies of all 64 possible trinucleotides. For each genome, we computed the colibactin-linked skewness metric by calculating the ratio of sum frequencies of the complementary trinucleotides ATA and TAT relative to the sum frequencies of the complementary trinucleotides AAA and TTT (Skew = (N_ATA_ + N_TAT_)/(N_AAA_ + N_TTT_). It is important to note that the nucleotide compositions on the trinucleotides in the numerator and denominator are equal (three A's and three T's). Therefore, our skewness measurement reflects bias in nucleotide order and is indifferent to differences in nucleotide composition. We classified strains as colibactin producers by the proteome annotation associated with each genome assembly. Strains harboring more than nine proteins annotated with “colibactin” in their description were classified as pks^+^ strains. Our choice of at least nine pks genes (∼50% of the pks genes) was used to account for potential missing coverage of the pks genes in the deposited genome sequences. Of the 19 genes in the island, 17 are considered essential for colibactin expression (Nougayrède et al. 2006). Out of the deposited genomes we analyzed, 794 had more than nine pks genes, and 98% of these (777) had 17 or more pks genes. Out of a total of 9089 *E. coli* genomes we analyzed, 794 strains were classified as pks^+^, with 98% (n = 777) of them belonging to the B2 phylogroup. The remaining pks^+^ strains belonged to the E (n = 2), A (n = 9), and B1 (n = 6) phylogroups. The B2 phylogroup is known to harbor the vast majority of pks^+^ strains in *E. coli* (Nougayrède et al. 2006; Wami et al. 2021).

## Data access

All raw sequencing data from the barcoded knockout library screen and whole genomes from the mutation accumulation experiment have been submitted to the NCBI BioProject database (https://www.ncbi.nlm.nih.gov/bioproject/) under accession numbers PRJNA1061230, PRJNA1060772, and PRJNA1060778. Code for all analyses can be found at GitHub (https://github.com/Mitchell-SysBio/2023_colibactin), Zenodo (https://doi.org/10.5281/zenodo.10419723), and as Supplemental Code.

## Supplementary Material

Supplement 1

Supplement 2

Supplement 3

Supplement 4

Supplement 5

Supplement 6

Supplement 7

Supplement 8

## References

[GR279517LOWC1] Abram K, Udaondo Z, Bleker C, Wanchai V, Wassenaar TM, Robeson MS, Ussery DW. 2021. Mash-based analyses of *Escherichia coli* genomes reveal 14 distinct phylogroups. Commun Biol 4: 117. 10.1038/s42003-020-01626-5 33500552 PMC7838162

[GR279517LOWC2] Bailey TL. 2021. STREME: accurate and versatile sequence motif discovery. Bioinformatics 37: 2834–2840. 10.1093/bioinformatics/btab203 33760053 PMC8479671

[GR279517LOWC3] Bossuet N, Guyonnet C, Chagneau CV, Tang-Fichaux M, Penary M, Loubet D, Branchu P, Oswald E, Nougayrede J-P. 2023. Oxygen concentration modulates colibactin production. Gut Microbes 15: 2222437. 10.1080/19490976.2023.2222437 37312436 PMC10269391

[GR279517LOWC4] Bossuet-Greif N, Dubois D, Petit C, Tronnet S, Martin P, Bonnet R, Oswald E, Nougayrède J-P. 2016. *Escherichia coli* ClbS is a colibactin resistance protein. Mol Microbiol 99: 897–908. 10.1111/mmi.13272 26560421

[GR279517LOWC5] Bossuet-Greif N, Vignard J, Taieb F, Mirey G, Dubois D, Petit C, Oswald E, Nougayrède J-P. 2018. The colibactin genotoxin generates DNA interstrand cross-links in infected cells. mBio 9: e02393-17. 10.1128/mBio.02393-17 29559578 PMC5874909

[GR279517LOWC6] Brotherton CA, Balskus EP. 2013. A prodrug resistance mechanism is involved in colibactin biosynthesis and cytotoxicity. J Am Chem Soc 135: 3359–3362. 10.1021/ja312154m 23406518

[GR279517LOWC7] Buc E, Dubois D, Sauvanet P, Raisch J, Delmas J, Darfeuille-Michaud A, Pezet D, Bonnet R. 2013. High prevalence of mucosa-associated *E. coli* producing cyclomodulin and genotoxin in colon cancer. PLoS One 8: e56964. 10.1371/journal.pone.0056964 23457644 PMC3572998

[GR279517LOWC8] Chagneau CV, Garcie C, Bossuet-Greif N, Tronnet S, Brachmann AO, Piel J, Nougayrède J-P, Martin P, Oswald E. 2019. The polyamine spermidine modulates the production of the bacterial genotoxin colibactin. mSphere 4: e00414-19. 10.1128/msphere.00414-19 31578245 PMC6796968

[GR279517LOWC9] Chen J, Byun H, Liu R, Jung I-J, Pu Q, Zhu CY, Tanchoco E, Alavi S, Degnan PH, Ma AT, 2022. A commensal-encoded genotoxin drives restriction of *Vibrio cholerae* colonization and host gut microbiome remodeling. Proc Natl Acad Sci 119: e2121180119. 10.1073/pnas.2121180119 35254905 PMC8931321

[GR279517LOWC10] Chen B, Ramazzotti D, Heide T, Spiteri I, Fernandez-Mateos J, James C, Magnani L, Graham TA, Sottoriva A. 2023. Contribution of pks^+^ *E. coli* mutations to colorectal carcinogenesis. Nat Commun 14: 7827. 10.1038/s41467-023-43329-5 38030613 PMC10687070

[GR279517LOWC11] Cole RS. 1973. Repair of DNA containing interstrand crosslinks in *Escherichia coli*: sequential excision and recombination. Proc Natl Acad Sci 70: 1064–1068. 10.1073/pnas.70.4.1064 4577788 PMC433426

[GR279517LOWC12] Cuevas-Ramos G, Petit CR, Marcq I, Boury M, Oswald E, Nougayrède J-P. 2010. *Escherichia coli* induces DNA damage in vivo and triggers genomic instability in mammalian cells. Proc Natl Acad Sci 107: 11537–11542. 10.1073/pnas.1001261107 20534522 PMC2895108

[GR279517LOWC13] Deatherage DE, Barrick JE. 2014. Identification of mutations in laboratory-evolved microbes from next-generation sequencing data using *breseq*. In Engineering and analyzing multicellular systems: methods and protocols (ed. Sun L, Shou W), pp. 165–188. Springer, New York. 10.1007/978-1-4939-0554-6_12 PMC4239701 24838886

[GR279517LOWC14] Dejea CM, Fathi P, Craig JM, Boleij A, Taddese R, Geis AL, Wu X, DeStefano Shields CE, Hechenbleikner EM, Huso DL, 2018. Patients with familial adenomatous polyposis harbor colonic biofilms containing tumorigenic bacteria. Science (1979) 359: 592–597. 10.1126/science.aah3648 PMC5881113 29420293

[GR279517LOWC15] Dougherty MW, Valdés-Mas R, Wernke KM, Gharaibeh RZ, Yang Y, Brant JO, Riva A, Muehlbauer M, Elinav E, Puschhof J, 2023. The microbial genotoxin colibactin exacerbates mismatch repair mutations in colorectal tumors. Neoplasia 43: 100918. 10.1016/j.neo.2023.100918 37499275 PMC10413156

[GR279517LOWC16] Dubinsky V, Dotan I, Gophna U. 2020. Carriage of colibactin-producing bacteria and colorectal cancer risk. Trends Microbiol 28: 874–876. 10.1016/j.tim.2020.05.015 32507544

[GR279517LOWC17] Dubois D, Delmas J, Cady A, Robin F, Sivignon A, Oswald E, Bonnet R. 2010. Cyclomodulins in urosepsis strains of *Escherichia coli*. J Clin Microbiol 48: 2122–2129. 10.1128/jcm.02365-09 20375237 PMC2884489

[GR279517LOWC18] Dubois D, Baron O, Cougnoux A, Delmas J, Pradel N, Boury M, Bouchon B, Bringer M-A, Nougayrède J-P, Oswald E, 2011. ClbP is a prototype of a peptidase subgroup involved in biosynthesis of nonribosomal peptides. J Biol Chem 286: 35562–35570. 10.1074/jbc.M111.221960 21795676 PMC3195562

[GR279517LOWC19] Dziubańska-Kusibab PJ, Berger H, Battistini F, Bouwman BAM, Iftekhar A, Katainen R, Cajuso T, Crosetto N, Orozco M, Aaltonen LA, 2020. Colibactin DNA-damage signature indicates mutational impact in colorectal cancer. Nat Med 26: 1063–1069. 10.1038/s41591-020-0908-2 32483361

[GR279517LOWC20] Eklöf V, Löfgren-Burström A, Zingmark C, Edin S, Larsson P, Karling P, Alexeyev O, Rutegård J, Wikberg ML, Palmqvist R. 2017. Cancer-associated fecal microbial markers in colorectal cancer detection. Int J Cancer 141: 2528–2536. 10.1002/ijc.31011 28833079 PMC5697688

[GR279517LOWC21] The Gene Ontology Consortium, Ashburner M, Ball CA, Blake JA, Botstein D, Butler H, Cherry JM, Davis AP, Dolinski K, Dwight SS, 2000. Gene Ontology: tool for the unification of biology. Nat Genet 25: 25–29. 10.1038/75556 10802651 PMC3037419

[GR279517LOWC22] Gibson DG, Young L, Chuang R-Y, Venter JC, Hutchison CA, Smith HO. 2009. Enzymatic assembly of DNA molecules up to several hundred kilobases. Nat Methods 6: 343–345. 10.1038/nmeth.1318 19363495

[GR279517LOWC23] Grant CE, Bailey TL, Noble WS. 2011. FIMO: scanning for occurrences of a given motif. Bioinformatics 27: 1017–1018. 10.1093/bioinformatics/btr064 21330290 PMC3065696

[GR279517LOWC24] Iyadorai T, Mariappan V, Vellasamy KM, Wanyiri JW, Roslani AC, Lee GK, Sears C, Vadivelu J. 2020. Prevalence and association of *pks*^+^ *Escherichia coli* with colorectal cancer in patients at the University Malaya Medical Centre, Malaysia. PLoS One 15: e0228217. 10.1371/journal.pone.0228217 31990962 PMC6986756

[GR279517LOWC25] Kanehisa M, Goto S. 2000. KEGG: Kyoto Encyclopedia of Genes and Genomes. Nucleic Acids Res 28: 27–30. 10.1093/nar/28.1.27 10592173 PMC102409

[GR279517LOWC26] Kern L, Abdeen SK, Kolodziejczyk AA, Elinav E. 2021. Commensal inter-bacterial interactions shaping the microbiota. Curr Opin Microbiol 63: 158–171. 10.1016/j.mib.2021.07.011 34365152

[GR279517LOWC27] Kowalczykowski SC, Dixon DA, Eggleston AK, Lauder SD, Rehrauer WM. 1994. Biochemistry of homologous recombination in *Escherichia coli*. Microbiol Rev 58: 401–465. 10.1128/mr.58.3.401-465.1994 7968921 PMC372975

[GR279517LOWC28] Lioy VS, Cournac A, Marbouty M, Duigou S, Mozziconacci J, Espéli O, Boccard F, Koszul R. 2018. Multiscale structuring of the *E. coli* chromosome by nucleoid-associated and condensin proteins. Cell 172: 771–783.e18. 10.1016/j.cell.2017.12.027 29358050

[GR279517LOWC29] Love MI, Huber W, Anders S. 2014. Moderated estimation of fold change and dispersion for RNA-seq data with DESeq2. Genome Biol 15: 550. 10.1186/s13059-014-0550-8 25516281 PMC4302049

[GR279517LOWC30] Luo W, Friedman MS, Shedden K, Hankenson KD, Woolf PJ. 2009. GAGE: generally applicable gene set enrichment for pathway analysis. BMC Bioinformatics 10: 161. 10.1186/1471-2105-10-161 19473525 PMC2696452

[GR279517LOWC31] Maslowska KH, Makiela-Dzbenska K, Fijalkowska IJ. 2019. The SOS system: a complex and tightly regulated response to DNA damage. Environ Mol Mutagen 60: 368–384. 10.1002/em.22267 30447030 PMC6590174

[GR279517LOWC32] Noto Guillen M, Rosener B, Sayin S, Mitchell A. 2021. Assembling stable syntrophic *Escherichia coli* communities by comprehensively identifying beneficiaries of secreted goods. Cell Syst 12: 1064–1078.e7. 10.1016/j.cels.2021.08.002 34469744 PMC8602757

[GR279517LOWC33] Noto Guillen M, Li C, Rosener B, Mitchell A. 2024. Antibacterial activity of nonantibiotics is orthogonal to standard antibiotics. Science 384: 93–100. 10.1126/science.adk7368 38484036 PMC12055234

[GR279517LOWC34] Nougayrède J-P, Homburg S, Taieb F, Boury M, Brzuszkiewicz E, Gottschalk G, Buchrieser C, Hacker J, Dobrindt U, Oswald E. 2006. *Escherichia coli* induces DNA double-strand breaks in eukaryotic cells. Science 313: 848–851. 10.1126/science.1127059 16902142

[GR279517LOWC35] Oliero M, Calvé A, Fragoso G, Cuisiniere T, Hajjar R, Dobrindt U, Santos MM. 2021. Oligosaccharides increase the genotoxic effect of colibactin produced by *pks+ Escherichia coli* strains. BMC Cancer 21: 172. 10.1186/s12885-021-07876-8 33596864 PMC7890614

[GR279517LOWC36] Pagès V, Koffel-Schwartz N, Fuchs RPP. 2003. *RecX*, a new SOS gene that is co-transcribed with the *recA* gene in *Escherichia coli*. DNA Repair (Amst) 2: 273–284. 10.1016/s1568-7864(02)00217-3 12547390

[GR279517LOWC37] Pleguezuelos-Manzano C, Puschhof J, Rosendahl Huber A, van Hoeck A, Wood HM, Nomburg J, Gurjao C, Manders F, Dalmasso G, Stege PB, 2020. Mutational signature in colorectal cancer caused by genotoxic *pks*^+^ *E. coli*. Nature 580: 269–273. 10.1038/s41586-020-2080-8 32106218 PMC8142898

[GR279517LOWC38] Ponder RG, Fonville NC, Rosenberg SM. 2005. A switch from high-fidelity to error-prone DNA double-strand break repair underlies stress-induced mutation. Mol Cell 19: 791–804. 10.1016/j.molcel.2005.07.025 16168374

[GR279517LOWC39] Reuter C, Alzheimer M, Walles H, Oelschlaeger TA. 2018. An adherent mucus layer attenuates the genotoxic effect of colibactin. Cell Microbiol 20: e12812. 10.1111/cmi.12812 29156489

[GR279517LOWC40] Rosener B, Sayin S, Oluoch PO, García González AP, Mori H, Walhout AJM, Mitchell A. 2020. Evolved bacterial resistance against fluoropyrimidines can lower chemotherapy impact in the *Caenorhabditis elegans*. eLife 9: e59831. 10.7554/eLife.59831 33252330 PMC7725501

[GR279517LOWC41] Salgado H, Gama-Castro S, Lara P, Mejia-Almonte C, Alarcón-Carranza G, López-Almazo AG, Betancourt-Figueroa F, Peña-Loredo P, Alquicira-Hernández S, Ledezma-Tejeida D, 2024. RegulonDB v12.0: a comprehensive resource of transcriptional regulation in *E. coli* K-12. Nucleic Acids Res 52: D255–D264. 10.1093/nar/gkad1072 37971353 PMC10767902

[GR279517LOWC42] Sayin S, Rosener B, Li CG, Ho B, Ponomarova O, Ward DV, Walhout AJM, Mitchell A. 2023. Evolved bacterial resistance to the chemotherapy gemcitabine modulates its efficacy in co-cultured cancer cells. eLife 12: e83140. 10.7554/eLife.83140 36734518 PMC9931390

[GR279517LOWC43] Shee C, Gibson JL, Darrow MC, Gonzalez C, Rosenberg SM. 2011. Impact of a stress-inducible switch to mutagenic repair of DNA breaks on mutation in *Escherichia coli*. Proc Natl Acad Sci 108: 13659–13664. 10.1073/pnas.1104681108 21808005 PMC3158223

[GR279517LOWC44] Silpe JE, Wong JWH, Owen SV, Baym M, Balskus EP. 2022. The bacterial toxin colibactin triggers prophage induction. Nature 603: 315–320. 10.1038/s41586-022-04444-3 35197633 PMC8907063

[GR279517LOWC45] Stohl EA, Brockman JP, Burkle KL, Morimatsu K, Kowalczykowski SC, Seifert HS. 2003. *Escherichia coli* RecX inhibits RecA recombinase and coprotease activities in vitro and in vivo. J Biol Chem 278: 2278–2285. 10.1074/jbc.M210496200 12427742

[GR279517LOWC46] Tripathi P, Shine EE, Healy AR, Kim CS, Herzon SB, Bruner SD, Crawford JM. 2017. ClbS is a cyclopropane hydrolase that confers colibactin resistance. J Am Chem Soc 139: 17719–17722. 10.1021/jacs.7b09971 29112397 PMC6202678

[GR279517LOWC47] Tronnet S, Garcie C, Rehm N, Dobrindt U, Oswald E, Martin P. 2016. Iron homeostasis regulates the genotoxicity of *Escherichia coli* that produces colibactin. Infect Immun 84: 3358–3368. 10.1128/iai.00659-16 27620723 PMC5116714

[GR279517LOWC48] Tronnet S, Garcie C, Brachmann AO, Piel J, Oswald E, Martin P. 2017. High iron supply inhibits the synthesis of the genotoxin colibactin by pathogenic *Escherichia coli* through a non-canonical Fur/RyhB-mediated pathway. Pathog Dis 75: ftx066. 10.1093/femspd/ftx066 28637194

[GR279517LOWC49] Velilla JA, Volpe MR, Kenney GE, Walsh RM, Balskus EP, Gaudet R. 2023. Structural basis of colibactin activation by the ClbP peptidase. Nat Chem Biol 19: 151–158. 10.1038/s41589-022-01142-z 36253550 PMC9889268

[GR279517LOWC50] Vizcaino MI, Crawford JM. 2015. The colibactin warhead crosslinks DNA. Nat Chem 7: 411–417. 10.1038/nchem.2221 25901819 PMC4499846

[GR279517LOWC51] Vollmer AC, Belkin S, Smulski DR, Van Tyk TK, LaRossa RA. 1997. Detection of DNA damage by use of *Escherichia coli* carrying recA′::lux, uvrA′::lux, or alkA′::lux reporter plasmids. Appl Environ Microbiol 63: 2566–2571. 10.1128/aem.63.7.2566-2571.1997 9212407 PMC168554

[GR279517LOWC52] Wami H, Wallenstein A, Sauer D, Stoll M, von Bünau R, Oswald E, Müller R, Dobrindt U. 2021. Insights into evolution and coexistence of the colibactin- and yersiniabactin secondary metabolite determinants in enterobacterial populations. Microb Genom 7: 000577. 10.1099/mgen.0.000577 34128785 PMC8461471

[GR279517LOWC53] Wang X, Kim Y, Ma Q, Hong SH, Pokusaeva K, Sturino JM, Wood TK. 2010. Cryptic prophages help bacteria cope with adverse environments. Nat Commun 1: 147. 10.1038/ncomms1146 21266997 PMC3105296

[GR279517LOWC54] Watanabe D, Murakami H, Ohno H, Tanisawa K, Konishi K, Tsunematsu Y, Sato M, Miyoshi N, Wakabayashi K, Watanabe K, 2020. Association between dietary intake and the prevalence of tumourigenic bacteria in the gut microbiota of middle-aged Japanese adults. Sci Rep 10: 15221. 10.1038/s41598-020-72245-7 32939005 PMC7495490

[GR279517LOWC55] Wilson MR, Jiang Y, Villalta PW, Stornetta A, Boudreau PD, Carrá A, Brennan CA, Chun E, Ngo L, Samson LD, 2019. The human gut bacterial genotoxin colibactin alkylates DNA. Science 363: eaar7785. 10.1126/science.aar7785 30765538 PMC6407708

[GR279517LOWC56] Wong JJ, Ho FK, Choo PY, Chong KKL, Ho CMB, Neelakandan R, Keogh D, Barkham T, Chen J, Liu CF, 2022. *Escherichia coli* BarA-UvrY regulates the pks island and kills Staphylococci via the genotoxin colibactin during interspecies competition. PLoS Pathog 18: e1010766. 10.1371/journal.ppat.1010766 36067266 PMC9481169

[GR279517LOWC57] Xue M, Shine E, Wang W, Crawford JM, Herzon SB. 2018. Characterization of natural colibactin–nucleobase adducts by tandem mass spectrometry and isotopic labeling. Support for DNA alkylation by cyclopropane ring opening. Biochemistry 57: 6391–6394. 10.1021/acs.biochem.8b01023 30365310 PMC6997931

[GR279517LOWC58] Xue M, Kim CS, Healy AR, Wernke KM, Wang Z, Frischling MC, Shine EE, Wang W, Herzon SB, Crawford JM. 2019. Structure elucidation of colibactin and its DNA cross-links. Science 365: eaax2685. 10.1126/science.aax2685 31395743 PMC6820679

[GR279517LOWC59] Zhai Y, Minnick PJ, Pribis JP, Garcia-Villada L, Hastings PJ, Herman C, Rosenberg SM. 2023. ppGpp and RNA-polymerase backtracking guide antibiotic-induced mutable gambler cells. Mol Cell 83: 1298–1310.e4. 10.1016/j.molcel.2023.03.003 36965481 PMC10317147

