# Supplementary material for: Colibactin leads to a bacteria-specific mutation pattern and self-inflicted DNA damage
Source: Genome Res. 2024 Aug;34(8):1154–64. doi: 10.1101/gr.279517.124 (PMC11444178; doi:10.1101/gr.279517.124)
Supplement: Supplement 1 [file supplemental_methods.pdf]

Genomic deletion primers:

gspI_del_carb_fw	tgccagaactggacgtgttttcctcgccgaatgaatcttgtgactgaagcGGCTAATGCACCCAGTAAGG
gpsI_del_carb_rv	tcaaacgctcgccagagatacccgcccatgaacaaacaatcagggatgacACTAGCAACACCAGAACAGCC
clbN_del_chl_fw	ttgtattccttaagggttggcatcgtgtttatagtgtccGCGGTATCATCAACAGGCTT
clbN_del_chl_rv	acaggaacagtgccacatcattgatcaactgtatccttacACGAAAGGGCCTCGTGATAC
clbS_del_chl_fw	gtccacactcatcgctgcccggaaaaatcatcagtggggaggcaaacggtaagcaccccgAGCGGTATCATCAACAGGCTT
clbS_del_chl_rv	ttgaatcactcgaaatagtagggtagaaatagttgtgtaactatacaaggagcaatagatACGAAAGGGCCTCGTGATAC

Note: lowercase letters are homology arms for genomic regions flanking the gene of interest; uppercase arms are primers for antibiotic resistance cassettes in plasmids.

Fluorescent DNA damage reporter primer sequences:

backbone_EM7_fw	GTTGACAATTAATCATCGGCATAGTATATCGGCATAGTATAATACGACGAATTCATTAAAGAGGAGAAAGG
backbone_recA_rev	GTAGAAATTGTTGCCACAAGATGGTTTCTTAGACGTCCAT
recA_backbone_fw	ATGGACGTCTAAGAAACCATCTTGTGGCAACAATTTCTAC
recA_YFP_rev	TTCTCCTCTTTAATGAATTCGATAGTCAATATGTTCTGTT
YFP_recA_fw	AACAGAACATATTGACTATCGAATTCATTAAAGAGGAGAA
YFP_EM7_rev	GCCGATGATTAATTGTCAACTCTAGGGCGGCGGATTTGTC

Backbone includes CFP and spectinomycin resistance
