# Supplementary material for: Colibactin leads to a bacteria-specific mutation pattern and self-inflicted DNA damage
Source: Genome Res. 2024 Aug;34(8):1154–64. doi: 10.1101/gr.279517.124 (PMC11444178; doi:10.1101/gr.279517.124)
Supplement: Supplement 2 [file Supplemental_Code.zip › Supplemental_Code/code/ReadMe.docx]

**Figures 1 and 2**

ASKA_lookup_map.mat is an input for fastq2barcodeCounts_v3_1.m and contains all knockout strain barcodes and strain indentifier information.

fastq2barcodeCounts_v3_1.m generates knockout barcode counts from fastq files. The script iterates over fastq files, removes reads with low quality scores, and searches each read for known barcodes (provided in ASKA_lookup_map.mat). Identified barcodes are summed and normalized to reads per million for each sample.

countsOddPlusEven.csv is an example barcoded knockout strain counts file generated by fastq2barcodeCounts_v3_1.m and is an input to BarcodeScreen.R

barcodeAnalysis.R is a function (used in BarcodeScreen.R) that runs DESeq and pathway enrichment anaylsis on barcoded screen fastq files. DESeq is used to determine p-values and fold-change of knockout strains in control and treatment samples. Outputs include hits, volcano plots, and pathway enrichment. It relies on outputs from fastq2barcodeCounts_v3_1.m

BarcodeScreen.R is a wrapper script using the barcodeAnalysis.R function to analyze barcoded knockout strain screens. Different comparisons of treatment and control groups can be assigned in this script.

comparison_pksP2pksN.csv is an example comparison file that can be input to BarcodeScreen.R

metadata_pksP2pksN.csv is an example metadata file to be input to BarcodeScreen.R to assign samples to control or treatment groups.

EcoCyc Pathways Entrez GMT.csv is a file input to BarcodeScreen.R that contains EcoCyc pathways and the entrez ID for genes in those pathways.

COG function Entrez GMT.csv is a file input to BarcodeScreen.R that contains COG pathways and the entrez ID for genes in those pathways.

**Figure 3**

calcMutationTable_v2.m is a script that filters mutations by certain criteria and removes mutations shared by both treatment and control groups. The output is a .txt file with condition, mutation position, and mutation sequence context. Other outputs are fasta format files of mutation sequence context for both treatment and control, and a randomly generated control.

writeMasterMutationList.m is a script that takes a breseq output, cleans up some of the columns, and adds information like mutation type or sequence context.

Breseq_mutation_list.xlsx is an example output from breseq that is an input to writeMasterMutationList.m

mutationType.m is a script that extracts types of mutations (i.e. sbs, small/medium/large indels) and the frequency of each mutation.

master_mutation_final.xlsx is a file containing cleaned up breseq results and extra information such as sequence context and mutation type.

ctrl_context.txt is the randomly generated control fasta file from calcMutationTable_v2.m to be used in downstream analysis with fimo.

pks+_context_5.txt is a fasta format file to be used in fimo and for downstream analysis identifying sequences that match the enriched motifs.

fimo.xlsx is the output from a fimo analysis run on mutation sequence context files (i.e. ctrl_context.txt and pks+_context_5.txt). it is an input to findSeqwithMotif.m

findSeqwithMotif.m is a script that finds mutated sequences that match the enriched motif identified with fimo. fimo.xlsx and pks+_context_5.txt are inputs.

plotMutPositions.m is a script that plots the genomic location of mutation positions on a circle plot and relative to macrodomains from macrodomain_positions.xlsx

mutationContext_5.txt is a file containing conditions, mutation positions, and mutation sequence context. It serves as an input to plotMutPositions.m

genomeFeatures.xlsx is a file containing positions of key genome features in E. coli such as the origin and terminus regions. It is an input to plotMutPositions.m

macrodomain_positions.xlsx is a file containing the end point positions of previously defined macrodomains. It is an input to plotMutPositions.m

**Figure 4**

backgroundSignal.mat holds background/autofluorescence of colonies on certain media at certain time points. These values are to be input to plot_colony_selfRecA.m to subtract from experimental values. Generated with plot_colonyBackground.m

plot_colonyBackground.m is a script that determines the background/autofluorescence of untagged colonies on different media agar plates over time for YFP and CFP. These values are saved in an output to be used in plot_colony_selfRecA.m

quant_backgroundSignal.m is a function used by plot_colonyBackground.m that segments colonies and measures fluorescent signal intensity

quant_selfRecASignal.m is a function used by plot_colony_selfRecA.m to segment colonies and measure YFP fluorescence.

plot_colony_selfRecA.m is a script that uses the quant_selfRecASignal.m function to quantify YFP expression in colonies inflicting self-damage. Background fluorescence of untagged colonies is subtracted from both YFP and CFP channels and then YFP is divided by CFP to normalize to overall protein levels in each colony

yfp.mat is an example output file from the quant_selfRecASignal.m function used in plot_colony_selfRecA.m. The file can also be used as an input for the plotting portion of plot_colony_selfRecA

countTriNucs.m is a script that iterates through all possible trinucleotide sequences and identifies the frequency of each sequence in downloaded genomes. Information for each genome is saved in dataTable_Ecoli.mat

dataTable_Ecoli.mat is an input to countTriNucs.m and contains information on over 9,000 E. coli genomes, including accession numbers.
