# Supplementary material for: Colibactin leads to a bacteria-specific mutation pattern and self-inflicted DNA damage
Source: Genome Res. 2024 Aug;34(8):1154–64. doi: 10.1101/gr.279517.124 (PMC11444178; doi:10.1101/gr.279517.124)
Supplement: Supplement 3 [file Supplemental_Figure_S1.pdf]

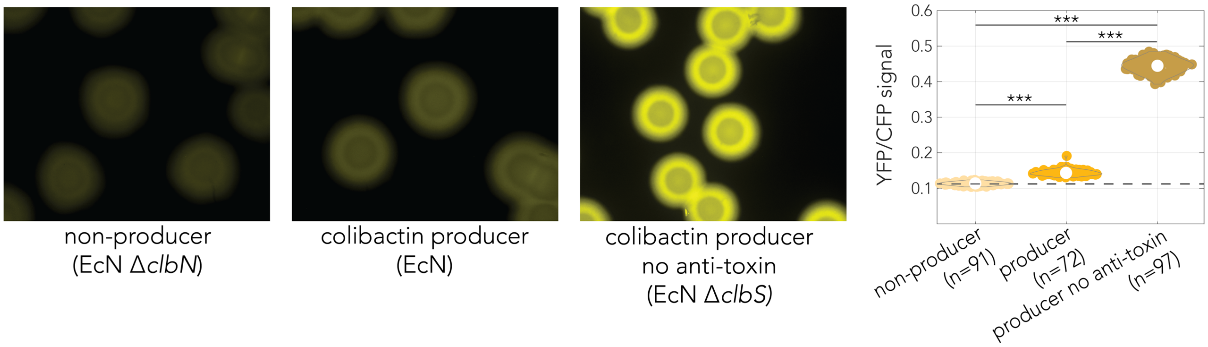

Supplementary Figure 1. Colibactin inflicts more self-damage in the absence of ClbS. Representative microscopy images of Nissle 1917 pks$^-$ (EcN Δ*clbN*), pks$^+$ (EcN), and pks$^+$ (EcN Δ*clbS*) expressing our *recA* reporter plasmid. Images show YFP expression, which represents *recA* activation in the colonies due to colibactin-induced self-damage. Fluorescence intensity range was set according to the intensity observed in the most highly damaged colibactin-producer (EcN Δ*clbS*) field of view. Violin plot displays the median YFP signal intensity per colony for each strain. Background YFP and CFP autofluorescence of the colonies was subtracted from each channel before YFP was normalized to CFP per colony (***$p<0.001$, two sample t-test).
